# Spatio-temporal effect of provincial technological innovation on environmental pollution in China

**DOI:** 10.3389/fpubh.2022.1073920

**Published:** 2022-11-24

**Authors:** Chu Wang, Xiaomin Guo

**Affiliations:** ^1^Business School, The University of Queensland, Brisbane, QLD, Australia; ^2^Institute of Geographic Sciences and Natural Resources Research, Chinese Academy of Sciences, Beijing, China; ^3^College of Resources and Environment, University of Chinese Academy of Sciences, Beijing, China

**Keywords:** technological innovation, environmental pollution, space effect, quantile regression, China

## Abstract

The relationship between technological innovation (TL) and environmental pollution (EP) and its action mechanisms are complex and controversial aspects of discussion. Using the spatial autocorrelation analysis, standard deviation ellipse analysis, kernel density function, spatial econometric model, this study analyzed the spatial distribution, evolution characteristics, and influencing factors of the EP and TL from 2000 to 2020 in China. Results found there was a significant spatial autocorrelation between the EP and TL in 2000–2020. The standard deviation ellipse of EP was broadly distributed in the “southwest-northeast” direction, indicating that EP presented a trend of concentration in the direction of “southwest-northeast.” The moving trajectory of the center of gravity for the EP in 2000–2020 was essentially moved from the northeast to southwest. Overall, the national level of TL exhibited a “north-south change, high in the east, and low in the west” trend. Regional differences were gradually expanding, and the polarization was evident. Regardless of using least squares method (OLS) or quantile regression (QR) models, TL, human capital (HC), and industrial structure (IS) all had an inhibitory effect on the EP at the effective significance level. Total population (TP), foreign direct investment (FDI), and local fiscal expenditure (LFE) were positively related to the EP.

## Introduction

With the rapidly development of global economy and industrialization, the global environmental sustainability is constantly threatened, which has aroused extensive attention (1, 2). Water pollution (3, 4), air pollution (*via* gaseous emissions) (5, 6), soil pollution (7), and air pollution (*via* other industrial pollutant emissions) have a significant influence on environmental stability (8). With the societal advancements, an awareness of the destruction of the natural environment has emerged (9–11). The book “Silent Spring” focuses on scientific information regarding environmental risks (12). Research has indicated that disparities in economic development levels could trap regions in environmental inequality problems such as environmental restriction policies (13), industrial structure layouts (14), and research & development (R&D) investment in green technology innovation (15, 16). Developed nations have a higher income level encouraging them to prioritize high-quality environmental development at an early stage. During the later stages of economic development, there was a progressive decline in environmental pollution as the economic development level increased (17). Environmental inequality intensifies the contradiction between economic development and pollution discharge in some regions, and key industrial emissions such as sulfur dioxide, soot, wastewater, and solid waste are significantly correlated in a spatial context (18–21). Although the geographical environment or climatic conditions influences environmental pollution, anthropogenic factors such as excessive use of fossil fuels, increased population densities, rapid urbanization, and industrialization remain the primary causes of environmental pollution and degradation (22–24).

Technological innovation (TL) is often considered the driving force behind economic growth, and it can play an important role in strengthening the competitiveness of enterprises and enhancing the national economy as a whole (25). Because of regional heterogeneity and asymmetry, TL has become a research hotspot for solving environmental pollution problems by developing a sustainable industrial structure (IS) (26). About the relationship between the environment and TL, during the previous studies, there is no research-based consensus on the relationship between TL and the environment pollution, with three main viewpoints existing. First, TL can effectively improve environmental quality in accordance with sustainable development goals (27). Second, TL can degrade environmental quality in the short term, but this trend can be reversed as an investment in core R&D technologies grows (28). Third, there is no influence or a non-linear relationship between TL and environmental quality (29). However, as research has progressed, the majority of researchers currently agree that TL is the most effective method to reduce environmental pollution under the guidance of reasonable policies. Despite the fact that TL cannot directly reduce the carbon emission intensity, it can indirectly reduce the carbon emission intensity by supporting the adjustment and optimization of IS, as demonstrated by a number of research findings (30). The industry-based economy has begun to minimize pollution through transformation and TL because of the progress in industrialization (31).

Researches concerning the reduction of environmental pollution by TL in the world are growing. Various studies have been conducted in domestic and foreign. Many international scholars have conducted research in succession (32–34). Such as, Mughal et al. (35) analyzed the panel data of five South Asian countries (Bangladesh, Bhutan, India, Maldives, and Pakistan) from 1990 to 2019 to examine the impacts of TL, EP, energy consumption, and economic growth in these countries. The results demonstrated that the development of TL within the context of policies on sustainable development had eased the consumption of energy, but the resultant consumption levels were still significant. Omri and Hadj (36) examined data from 23 emerging economies and found that most developing countries could effectively reduce carbon dioxide emissions through TL and solid environmental governance. Iqbal et al. (37) used data on the consumption of renewable energy in 37 Organization for Economic Co-operation and Development (OECD) member countries and the enhanced mean group (AMG) method to examine and conclude that renewable energy and TL could contribute to environmental improvement.

As the largest developing country globally, the rapid industrialization and high-quality economic development of China have led to an increase in its income level and heightened concern for EP. Chen et al. (38) analyzed the impact of enterprise TL on air pollution using large enterprise-level data sets from 1998 to 2012. The results showed that TL could significantly reduce the pollution from emissions of enterprises. The sulfur dioxide emissions of industries could be reduced by 2.71% if patent authorizations were increased by 1%. Following further analysis, it was determined that the industrial sector, geographical location, and ownership type influenced the reduction in air pollution *via* TL. Hao et al. (29) examined the impact of TL on EP from the perspective of the quantity and quality of foreign direct investment (FDI) using panel data between 2006 and 2016 for 30 Chinese provinces. The findings revealed that there was a complex non-linear relationship between TL capacity and environmental pollution. When the level of FDI increased from a low to a high level, TL could improve the quality of the environment. However, when the level of FDI went beyond a certain threshold, this beneficial impact would diminish. The ability of TL to have a positive impact on environmental pollution may improve as the quality of FDI grew. Xin and Lv (39) utilized a geographically weighted regression model to analyze the regional differences and impact mechanisms of TL on environmental pollution using data from 285 cities in China. The results indicated that both TL and environmental pollution exhibited significant spatial agglomeration, with urban TL having a detrimental effect on environmental pollution. Economic development, human capital, FDI, environmental regulation, and other factors jointly affected the pollution improvement effect of TL, which indirectly reduced environmental pollution by optimizing IS *via* technological progress.

Therefore, it can be deduced that besides TL, there are also many other factors have the most significant effects, such as the acceleration of economic development, the modification of IS, the improvement of environmental pollution-control policies. Therefore, the impact of TL on EP is unpredictable and limited by numerous factors, and different regions have distinct geographical characteristics. Existing studies have discussed the mechanism of TL affecting EP, but most of them are qualitative analysis. There is a lack of quantitative research on the degree of interaction among various influencing factors at the spatial level.

In view of the research deficiency above, this study aimed to analyze the interaction between TL and EP by focusing on 31 provinces in China, taking into account various driving factors, such as TP, GDP, IS, FDI, HC, ERS, and LFE among other factors. By analyzing this mechanism, this study could facilitate a better understanding of the specific mechanisms of inter-provincial TL capacity and environmental pollution and provide a theoretical basis for the Chinese government to formulate innovative incentive policies and environmental pollution control measures that are reasonable and effective. Moreover, it served as a scientific reference for coordinating the development capacity of TL between provinces and enhancing environmental governance.

## Data sources and variables selection

The sample interval of this study was set as 2000–2020, owing to data availability, and the total scope of the research covered 31 provinces (including municipalities and autonomous regions, excluding Hong Kong, Macao, and Taiwan). In view of the previous researches (39–41), based on the principles of objectivity, impartiality and data availability, on the premise that the explanatory variable and the explained variable are determined, this study selected the main control variables. Data sources of the variables can been in Table 1.

**Table 1:** Variable setting.

**Variable type**	**Variable definition**	**Variable description**	**Data source**
Explained variable	Environmental pollution (EP)	Comprehensive index of industrial wastewater discharge, industrial SO_2_ discharge, and industrial smoke (powder) dust discharge	“China Statistical Yearbook,” statistical yearbook of each province
Explanatory variable	Technological innovation (TL)	Number of green invention patent applications	China national intellectual property administration (https://www.cnipa.gov.cn/)
Control variable	Total population (TP)	Total regional population	“China statistical yearbook”
	Gross domestic product (GDP)	Regional gross domestic product	“China statistical yearbook”
	Human capital (HC)	Number of college students per 10,000 people	“China statistical yearbook”
	Foreign direct investment (FDI)	Regional foreign direct investment	“China statistical yearbook”
	Ratio of added value of secondary and tertiary industries (IS)	Added value of secondary industry/added value of tertiary industry	“China statistical yearbook”
	Environmental regulation (ERS)	The intensity of environmental regulation is measured by the comprehensive index of expenditure and regulatory indicators	“China statistical yearbook,” “China environmental statistics yearbook”
	Local fiscal expenditure (LFE)	Local fiscal expenditure	“China statistical yearbook”

### Explained variables

Environmental pollution (EP) index: environmental deterioration is mainly caused by industrial pollution. Numerous researches utilize industrial emission indicators to quantify the level of environmental pollution. Accordingly, this study selected three indicators, namely, industrial wastewater discharge, industrial SO_2_ discharge, and industrial smoke (powder) dust discharge, calculated their weights using the entropy method, and calculated a comprehensive index of environmental pollution for each province using weighted summation.

### Explanatory variables

Technological innovation level (TL): this study considered the number of invention patent authorizations as an indicator to measure the level of TL.

### Control variables

In addition to TL, other factors affect the ecological environment. The following control variables were selected in this study based on their comparability and the availability of data:

(1) Gross domestic product (GDP): the total GDP is a measure of the amount of economic development.

(2) Total regional population (TP): the TP of the region represents the population scale of the region.

(3) Industrial structure (IS): as an important link between human economic activities and the ecological environment, IS defines the types and quantities of pollution emissions and has varying degrees of impact on the ecological environment. Comparatively, the secondary industries have a far greater coercive effect on the ecological environment than primary and tertiary industries. However, owing to the advancement of information technology, the tertiary industry has gradually assumed a dominant position in the IS. This study measured the IS by the ratio of the added value of the secondary industry to that of the tertiary industry, taking into account the environmental impact of the secondary industry and the change in the IS.

(4) Foreign direct investment (FDI): as an important expression of economic globalization, it is debatable what impact FDI has on the environment, but its impact is undeniable. This study used the quantities of FDI to measure its environmental impact.

(5) Human capital (HC): human capital is the carrier of knowledge and technology, as well as an indispensable aspect for analyzing the environmental effects of HC. Once human capital attains a high level, HC can benefit the local environment *via* technology spillover. The academic community has not established a consensus on human capital measurement indicators. This study was constrained by the availability of provincial data provided references to prior research to measure the amount of human capital using the number of college students per 10,000 people.

(6) Environmental regulation (ERS): the majority of existing studies estimate the intensity of environmental regulation from the perspectives of environmental input and environmental performance. The former consists of pollution control investments, government environmental protection fiscal expenditures, and emission reduction costs, whereas the latter comprises pollution discharge fees, pollution discharge taxes, and pollutant disposal rates. This study covered economic-environmental regulation because it was simpler to internalize external environmental costs *via* economic-environmental regulation (42). Based on the consistency and availability of data, this study employed a comprehensive index of expenditure and regulatory indicators to assess the intensity of environmental regulation. The expenditure index focused on governance expenditures, which were measured by the ratio of industrial pollution control expenditures to industrial added value. The regulatory indicators are based on the monitoring of government departments in implementing the environmental regulation system and policies, following the practice of existing research (43, 44), and using the revenue from domestic sewage charges as the regulatory indicators. In this study, the min-max standardized method was used to calculate the comprehensive index of environmental regulation variables. The formula used was as follows:

(1) $$\begin{array}{l} ERS_{ij}=\frac{ERSM_{ij}-\min\left(ERSM_{i}\right)}{\max\left(ERSM_{i}\right)-\min\left(ERSM_{i}\right)}+\frac{ERRI_{ij}-\min\left(ERRI_{i}\right)}{\max\left(ERRI_{i}\right)-\min\left(ERRI_{i}\right)}\\ \;\left(i=1,2,\ldots ,21;j=1,2,\ldots ,31\right) \end{array}$$

Where *ERS*_*ij*_ represented the comprehensive index of environmental regulation of the *j*th province in the *i*th year, *ERSM*_*ij*_ and *ERRI*_*ij*_ were the proportions of investment in environmental regulation and the average income of environmental regulation of the *j*th province in the *i*th year, respectively. The max(*ERSM*_*i*_) and min(*ERSM*_*i*_) represented the maximum and minimum values of the proportion of environmental regulation investment in each province of the country in the *i*th year, respectively. The max(*ERRI*_*i*_) and min(*ERRI*_*i*_) represented the maximum and minimum of the average income of environmental regulation in each province of the country in the *i*th year, respectively.

(7) Local fiscal expenditure (LFE): the local financial expenditure was used to represent the level of concern for local financing of public goods.

## Methods

### Spatial autocorrelation analysis

(1) Global spatial autocorrelation. Spatial correlation of variables is the premise of using spatial econometric models. In this study, Moran's index (Moran's *I*) was used to test the spatial autocorrelation of core variables (45). If the sample range of the spatial correlation test was set to encompass the whole research scope of this study, the global Moran index could be utilized to reflect the spatial distribution pattern state of variables across the entire region (i.e., determining whether a clustering distribution existed). Formula (2) demonstrates the calculation method:

(2) $$\begin{array}{l} I=\frac{\overset{n}{\underset{i=1}{\sum }}\overset{n}{\underset{j=1}{\sum }}W_{ij}\left(Y_{ij}-\bar{Y}\right)\left(Y_{j}-\bar{Y}\right)}{S^{2}\overset{n}{\underset{i=1}{\sum }}\overset{n}{\underset{j=1}{\sum }}W_{ij}} \end{array}$$

where $S^{2}=\frac{\sum_{i=1}^{n}\left(Y_{i}-Ȳ\right)}{n}$ represented the sample variance, *W*_*ij*_ represented the spatial weight matrix, and *Y*_*i*_ and *Y*_*j*_ were the observed values of area *i* and area *j*, respectively. The value range of Moran's *I* index was −1 ≤Moran's *I* ≤1. If the *I* value was >0, it indicated a positive spatial autocorrelation; when the *I* value was <0, it indicated a negative spatial autocorrelation; when *I* was equal to 0, there was no spatial autocorrelation.

(2) Local spatial autocorrelation. Global Moran's index can only characterize the cluster status of core variables within a provincial scope and is incapable of accurately locating and distinguishing the specific spatial correlation patterns of the region (45). The local spatial correlation index LISA could describe whether the aggregation distribution between a region and its surrounding areas was a high- or low-value aggregation. The calculation method is shown in formula (3):

(3) $$\begin{array}{l} I_{i}=\frac{\left(Y_{i}-\bar{Y}\right)\overset{n}{\underset{j=1}{\sum }}W_{ij}\left(Y_{j}-\bar{Y}\right)}{S^{2}} \end{array}$$

where $S^{2}=\frac{\overset{n}{\underset{j=1,j\neq i}{\sum }}Y_{j}-\bar{Y}}{n-1}$; *I*_*i*_ is the local spatial autocorrelation coefficient of the *i*th province; *Y*_*i*_ represents the observation value of the *i*th province; and *W*_*ij*_ represents the spatial weight matrix. Based on the local Moran's *I* index, the LISA agglomeration map can be drawn up, which classifies the local spatial connection forms into High-High cluster, Low-Low cluster, Low-High cluster, and High-Low cluster.

### Standard deviation ellipse analysis

The standard deviation ellipse derived from spatial statistics can accurately reveal the spatial distribution characteristics of geographical elements (46). The model primarily represents the shape, orientation, distribution range, and other characteristics of the spatial distribution of attribute values of the research scope *via* the long axis, short axis, center of gravity, and rotational angle (47). The formula is as follows:

(4) $$\begin{array}{l} \bar{X_{w}}=\frac{\overset{n}{\underset{i=1}{\sum }}w_{i}x_{i}}{\overset{n}{\underset{i=1}{\sum }}w_{i}}\;\bar{Y_{w}}=\frac{\overset{n}{\underset{i=1}{\sum }}w_{i}y_{i}}{\overset{n}{\underset{i=1}{\sum }}w_{i}} \end{array}$$

(5) $$\begin{array}{l} \tan\theta =\frac{\left(\overset{n}{\underset{i=1}{\sum }}w_{i}^{2}\Delta x_{i}^{2}-\overset{n}{\underset{i=1}{\sum }}w_{i}^{2}\Delta y_{i}^{2}\right)+\sqrt{\overset{n}{\underset{i=1}{\sum }}w_{i}^{2}\Delta x_{i}^{2}-\overset{n}{\underset{i=1}{\sum }}w_{i}^{2}\Delta y_{i}^{2})+4\overset{n}{\underset{i=1}{\sum }}w_{i}^{2}\Delta x_{i}^{2}\Delta y_{i}^{2}}}{2\overset{n}{\underset{i=1}{\sum }}w_{i}^{2}\Delta x_{i}^{\;}\Delta y_{i}^{\;}} \end{array}$$

(6) $$\begin{array}{l} \sigma_{x}=\sqrt{\frac{\overset{n}{\underset{i=1}{\sum }}{\left(w_{i}\Delta x_{i}\cos\theta -w_{i}\Delta y_{i}\sin\theta \right)}^{2}}{\overset{n}{\underset{i=1}{\sum }}w_{i}^{2}}}\\ \sigma_{y}=\sqrt{\frac{\overset{n}{\underset{i=1}{\sum }}{\left(w_{i}\Delta x_{i}\sin\theta -w_{i}\Delta y_{i}\cos\theta \right)}^{2}}{\overset{n}{\underset{i=1}{\sum }}w_{i}^{2}}} \end{array}$$

(7) $$\begin{array}{l} S=\pi \sigma_{x}\sigma_{y} \end{array}$$

where (*x*_*i*_, *y*_*i*_) indicates the geographical coordinates of province *i* in the study area, and *w*_*i*_ represents the weight. (*X*_*w*_, *Y*_*w*_) represents the weighted average geographical center coordinates of the study area; θ is the azimuth angle of the ellipse (Δ*x*_*i*_, Δ*y*_*i*_); represents the deviation between the geographical location of province *i* in the study area and the weighted average geographical center coordinates of the study area; σ_*x*_ and σ_*v*_ represent the standard deviation values along the X-axis and Y-axis, respectively; and *S* is the area of the ellipse.

### Trend surface analysis

Trend surface analysis is based on spatial data and uses mathematical analysis methods to simulate spatial surfaces, depict the spatial distribution law of geographical elements, and consequently investigate the spatial change trend of geographical elements. It has significant utility in spatial analyses (48). In this study, we used coupled co-scheduling as the observation value, and we simulated the spatial differentiation characteristics of TL of provinces in China using trend surface analysis. If (*x*_*i*_, *y*_*i*_) is the spatial position of the *i*th province, then Z (*x*_*i*_, *y*_*i*_) represents the trend function of the *i*th province, where the X-axis depicts the east-west direction, and the Y-axis depicts the north-south direction.

### Dynamic evaluation analysis model

To further investigate the spatiotemporal dynamic characteristics of the EP, the kernel density function was employed to analyze its temporal characteristics. The kernel density function method analyzes the overall spatial change and evaluates the overall difference by adjusting the convergence degree and range of the function curve. The kernel density function is as follows:

(8) $$\begin{array}{l} f\left(x\right)\text{=}\frac{1}{nh}\overset{t}{\underset{i=1}{\sum }}K\left(\frac{x_{i}-x}{h}\right) \end{array}$$

where *n* is the total number of samples and *h* is the set window width, where *lim n* → ∞, *h*(*n*) = 0. In this study, the Gaussian kernel function is used for estimation, and its expression is:

(9) $$\begin{array}{l} K\left(x\right)=\frac{1}{\sqrt{2\pi }}\exp\left[-\frac{x^{2}}{2}\right] \end{array}$$

By combining the distribution form and the kernel density function diagram, we could effectively judge the change of the EP across various observational periods and then characterize its dynamic characteristics.

### The driving factors based on the quantile regression model

The least squares method is the most commonly used method of fitting a regression curve to given data (49). The basic notion is as follows:

(10) $$\begin{array}{l} f\left(x\right)=\alpha_{1}\varphi_{1}\left(x\right)+\alpha_{2}\varphi_{2}\left(x\right)+...+\alpha_{m}\varphi_{m}\left(x\right) \end{array}$$

where φ_*k*_(*x*) is a predetermined set of linearly independent functions; α_*k*_ is an undetermined coefficient (k = 1, 2, …, m, m < n); and the fitting criterion is to minimize the square sum of the distance $\partial_{i}^{\;}$ between *y*_*i*_ (*i* = 1, 2, …, n) and *f*(*x*_*i*_), also known as the least square criterion.

Traditional regression models emphasize the influence of explanatory variables on the conditional expectations of the explained variables, depicting a concentrated trend while frequently disregarding the coefficient changes resulting from the conditional random probability distribution. To remedy this flaw, Koenker and Bassett Jr. (50) developed the QR model. This model excludes the interference of outliers more effectively than other models and does presume that the data follow a normal distribution. It can examine the influence of explanatory variables on explained variables in different quartiles in an effective manner (51). The formula for the panel data is as follows:

(11) $$\begin{array}{l} Q_{Y_{it}}\left(\tau |X_{it}\right)=\alpha_{i}+{X_{it}}^{T}\beta \left(\tau \right),(i=1,2,\ldots ,n;\\ \;t=1,2,\ldots ,T) \end{array}$$

where *Q*_*Yit*_ is the conditional quantile function; *i* is the individual of different samples; *t* is the sample observation period; *n* is the sample size; *T* is the study period; τ is the quantile set in this study, and the value range is (0, 1); α_*i*_ is a constant term; and β(τ) is the influence coefficient at the τ quantile, which isoften estimated using weighted least squares β.

(12) $$\begin{array}{l} \beta \left(\theta \right)=\min_{\left(\alpha ,\beta \right)}\overset{q}{\underset{k=1}{\sum }}\overset{n}{\underset{i=1}{\sum }}\overset{T}{\underset{t=1}{\sum }}w_{k}\rho_{\tau_{k}}\left[Y_{it}-\alpha_{i}-X_{it}^{T}\beta \left(\tau_{k}\right)\right] \end{array}$$

where β(θ) represents the influence coefficient; *k* is the *k*th group of quantiles; *q* represents the number of quantile arrays; *w*_*k*_ is the weight coefficient of the *k* quantile; ρ_τ*k*_ represents the quantile loss function; and β(τ_*k*_) is the influence coefficient of the *k* quantile.

In order to clearly express regional differences, the Table 2 shows the specific divisions of the eastern, central and western provinces in China.

**Table 2:** Regional division of China.

**Region**	**Provinces**
Eastern China	Beijing, Tianjin, Shanghai, Liaoning, Jiangsu. Zhejiang, Fujian, Shandong, Guangdong.
Central China	Hebei, Shanxi, Jilin, Heilongjiang, Anhui, Jiangxi, Henan, Hubei, Hunan, Hainan.
Western China	Inner Mongolia, Guangxi, Chongqing, Sichuan, Guizhou, Yunnan, Xizang, Shaanxi, Gansu, Ningxia, Qinghai, Xinjiang.

## Results

### Spatial autocorrelation analysis

The global spatial autocorrelation, as measured by the global Moran's *I* index, can reflect the interdependence of core variables in various regions in the global range. Table 3 depicts Moran's *I* index of the spatial distribution of environmental pollution and TL under the conditions of a geographical adjacency matrix from 2000 to 2020. In the majority of provinces, the results indicated there is a significant spatial autocorrelation between EP and TL, which could be utilized for subsequent spatial effect measurements and estimation.

**Table 3:** Global Moran's *I* index of each core variable from 2000 to 2020.

**Year**	**Variable**	***I***	**Year**	**Variable**	***I***	**Year**	**Variable**	***I***
2000	TL	0.0980^**^	2007	TL	0.0064^*^	2014	TL	0.0077^*^
	EP	0.0281^*^		EP	0.7720^*^		EP	0.1229^**^
2001	TL	0.0442^**^	2008	TL	0.0375^*^	2015	TL	0.0264^**^
	EP	0.0387^*^		EP	0.0756^*^		EP	0.1012^**^
2002	TL	0.0382^**^	2009	TL	0.0456^*^	2016	TL	0.0340^**^
	EP	0.0403^*^		EP	0.0735^*^		EP	0.1051^**^
2003	TL	0.2299^*^	2010	TL	0.0449^*^	2017	TL	0.0012^*^
	EP	0.0574^*^		EP	0.0989^**^		EP	0.1298^**^
2004	TL	0.0504^*^	2011	TL	0.0394^*^	2018	TL	0.0033^*^
	EP	0.0725^*^		EP	0.1283^**^		EP	0.0789^*^
2005	TL	0.0054^*^	2012	TL	0.0249^*^	2019	TL	0.0241^*^
	EP	0.0651^*^		EP	0.1352^**^		EP	0.0606^*^
2006	TL	0.0092^*^	2013	TL	0.0125^*^	2020	TL	0.0069^*^
	EP	0.0628^*^		EP	0.1325^**^		EP	0.1090^**^

*, **, and *** Mean significant correlation at the level of 10%, 5%, and 1%, respectively.

**Figure 1:**
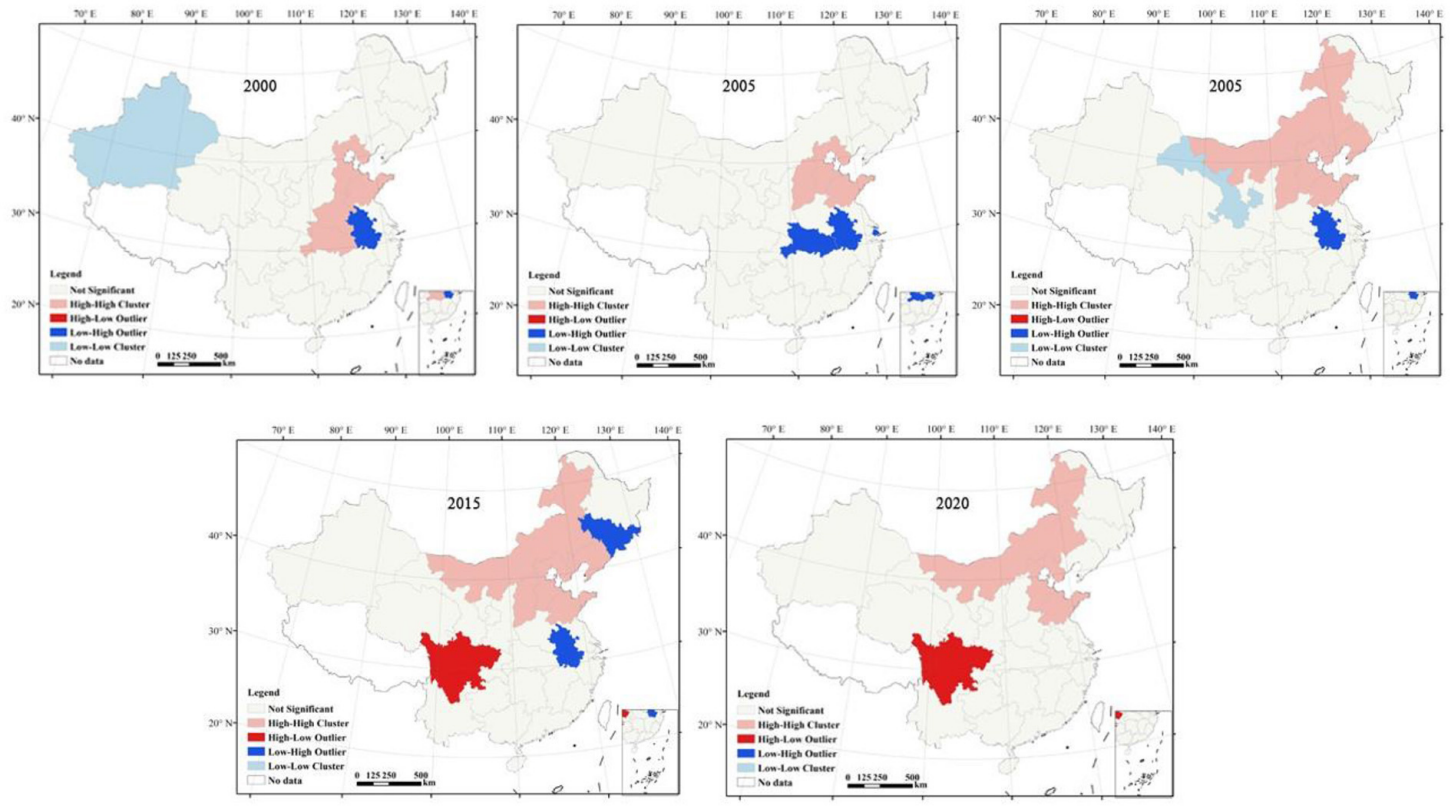
LISA cluster diagram of EP in 2000–2020.

For further analysis, this study further uses LISA scatterplot to characterize the EP clustering distribution of the area and its surrounding areas (Figure 1). Figure 1 shows that, except for insignificant areas, there were four spatial clustering types of EP: High-High cluster, Low-Low cluster, Low-High cluster, and High-Low cluster. Quantitatively, the positive spatial correlations of High-High cluster and Low-High cluster are predominated. Among them, from 2000 to 2020, High-High cluster of EP agglomerations are mainly distributed in the central and northern areas, which may be due to the fact that the technological innovation and industrialization of these cities are at a high level, and the negative impact of industrialization on the EP is greater than the positive effect of technological innovation, and technological innovation activities are not enough to improve the EP in the surrounding areas; Low-High cluster of EP agglomerations are mainly located in the central and southern areas of China (Hubei, Anhui), indicating that these areas have a low level of EP themselves and a high level of EP in the surrounding areas.

### Trend analysis

#### Trend analysis of core explanatory variables

**Figure 2:**
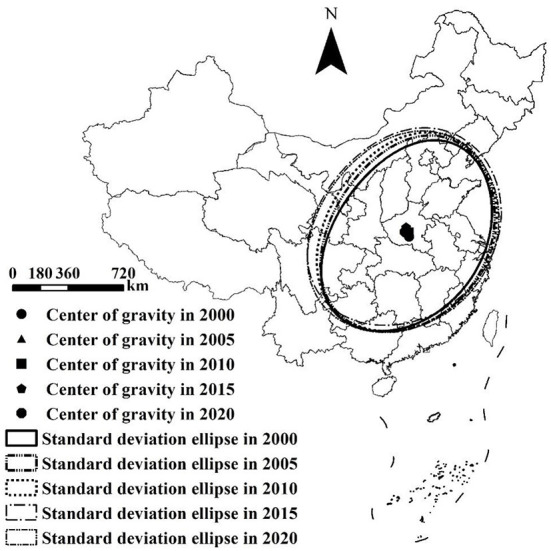
Standard deviation ellipse and center of gravity movement trajectory of China's environmental pollution index (EP) from 2000 to 2020.

The spatial distribution direction and dynamic characteristics of the comprehensive EP at the national and provincial scales were investigated using the standard deviation ellipse analysis tool of ArcGIS 10.3 software (Environmental Systems Research Institute), as listed in Table 4 and Figure 2.

**Table 4:** Basic information of standard deviation ellipse analysis.

**Year**	**Circumference of the ellipse (km)**	**Elliptical area (km^2^)**	**Longitude**	**Latitude**	**X-axis length (km)**	**Y-axis length (km)**	**Direction**
2000	58.23	255.86	113.46	33.11	7.47	10.91	56.53°
2005	59.75	274.33	113.55	33.31	8.02	10.89	57.30°
2010	61.24	289.14	113.45	33.50	8.30	11.10	60.05°
2015	63.87	313.05	113.20	34.00	8.54	11.67	68.54°
2020	63.77	314.11	113.01	33.55	8.68	11.52	65.95°

The results show that the following: (1) the standard deviation ellipse was generally positioned in the eastern China. The circumference of the ellipse showed a “first increasing and then decreasing” trend; it increased from 58.23 km in 2000 to 63.87 km in 2015 and then decreased to 63.77 km in 2020. (2) The area of the ellipse showed a “continuous increase” trend; it increased from 255.86 km^2^ in 2000 to 314.11 km^2^ in 2020, indicating that the EP of cities outside the ellipse was higher than that of cities inside the ellipse during 2000–2020. During the study period, the EP in the central region had a spatial distribution characteristic of “continuous expansion.” (3) There was a clear difference between the long and short axes of the standard deviation ellipse, indicating that the spatial distribution of the national EP was directional. The standard deviation ellipse was broadly distributed in the “southwest-northeast” direction, indicating that EP presents a trend of concentration in the direction of “southwest-northeast.” (4) The rotational angle of the standard deviation ellipse increased from 56.53° in 2000 to 68.54° in 2015 and then decreased to 65.95° in 2020. It revealed that the ellipse first rotated counterclockwise and subsequently slightly clockwise, indicating that the EP in the southwest or northeast changed rapidly. (5) The moving trajectory of the center of gravity of the EP from 2000 to 2020 was as follows: from 2000 to 2005, it moved to the northeast; from 2005 to 2015, it continued to move to the northwest; and from 2015 to 2020, it moved to the southwest. Henan Province was always the center of gravity of the EP from 2000 to 2020 (Figure 2).

**Figure 3:**
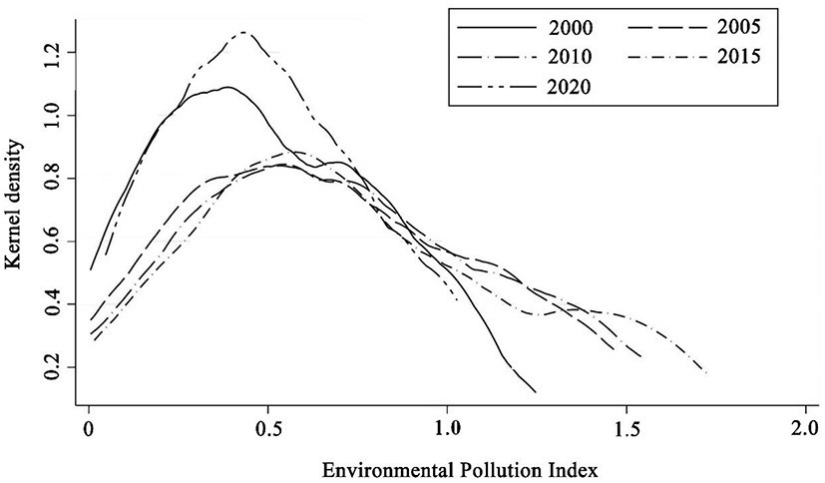
Dynamic evolution of the national EP from 2000 to 2020.

To better elucidate the dynamic characteristics of regional differences in the EP, this study used the Gaussian kernel density function and growth distribution chart to depict the evolution trend of regional differences in the EP. The data for 2000, 2005, 2010, 2015, and 2020 were selected, and the dynamic evolution process of the national and provincial EPs was plotted using Stata 15 software (Computer Resource Center) (see Figure 3).

Overall, the peak of the kernel density curve of the national and provincial EPs slowed down progressively. In 2005, the center of the density function shifted to the right compared to 2000, and the peak declined, indicating that the regional differences were increasing. In 2010 and 2015, the center of the density function shifted to the right by a substantial margin, the peak slowed, and the width significantly widened, indicating that the disparity between regions in the EP expanded with time. In 2020, the peak value of the kernel density function of the EP abruptly increased, and the center of the function shifted to the left, indicating that the polarization of the regional EP had weakened.

#### Trend analysis of explained variables

**Figure 4:**
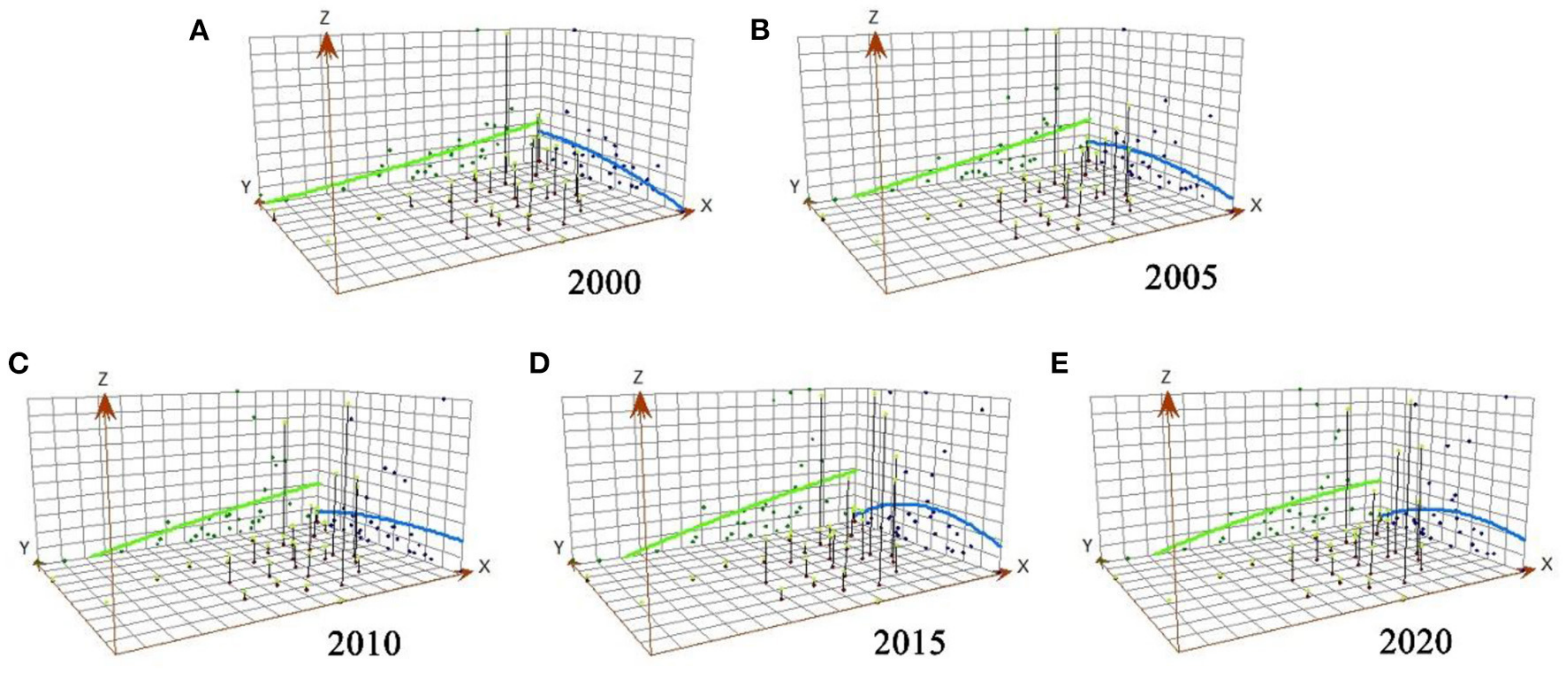
**(A–E)** Trend surface analysis results of provincial technological innovation (TL) levels in China.

The trend surface analysis was used to process and analyze the national TL level from 2000 to 2020 to analyze the changing trend and distribution law of TL at the national and provincial levels. The X-axis signifies the due east direction; the Y-axis represents the due north direction; the blue curve shows the change fitting line of the national TL in a north-south direction; and the green curve represents the change fitting line in an east-west direction (Figure 4). Figure 4 demonstrates that the national green technology innovation index in its entirety exhibits a trend of “north-south change, high in the east, and low in the west”.

Based on the north-south trend of the curve, the level of TL in 2000–2005 displayed a clear north-south spatial distribution, with high levels in the north and low levels in the south (Figures 4A,B). However, from 2010 to 2020, it gradually transformed into a significant spatial distribution characterized by low values in the north and high values in the south. The northeast showed evident spatial characteristics of “high in the east and low in the west.” While, the gap between the east and the west slightly widened, indicating that the gap of TL level at the provincial level in China was steadily widening in an east-west direction. The level of TL among regions had developed to varying degrees, and the regional gap was deepening (Figures 4C–E).

### Influencing factors of green technology innovation level

**Figure 5:**
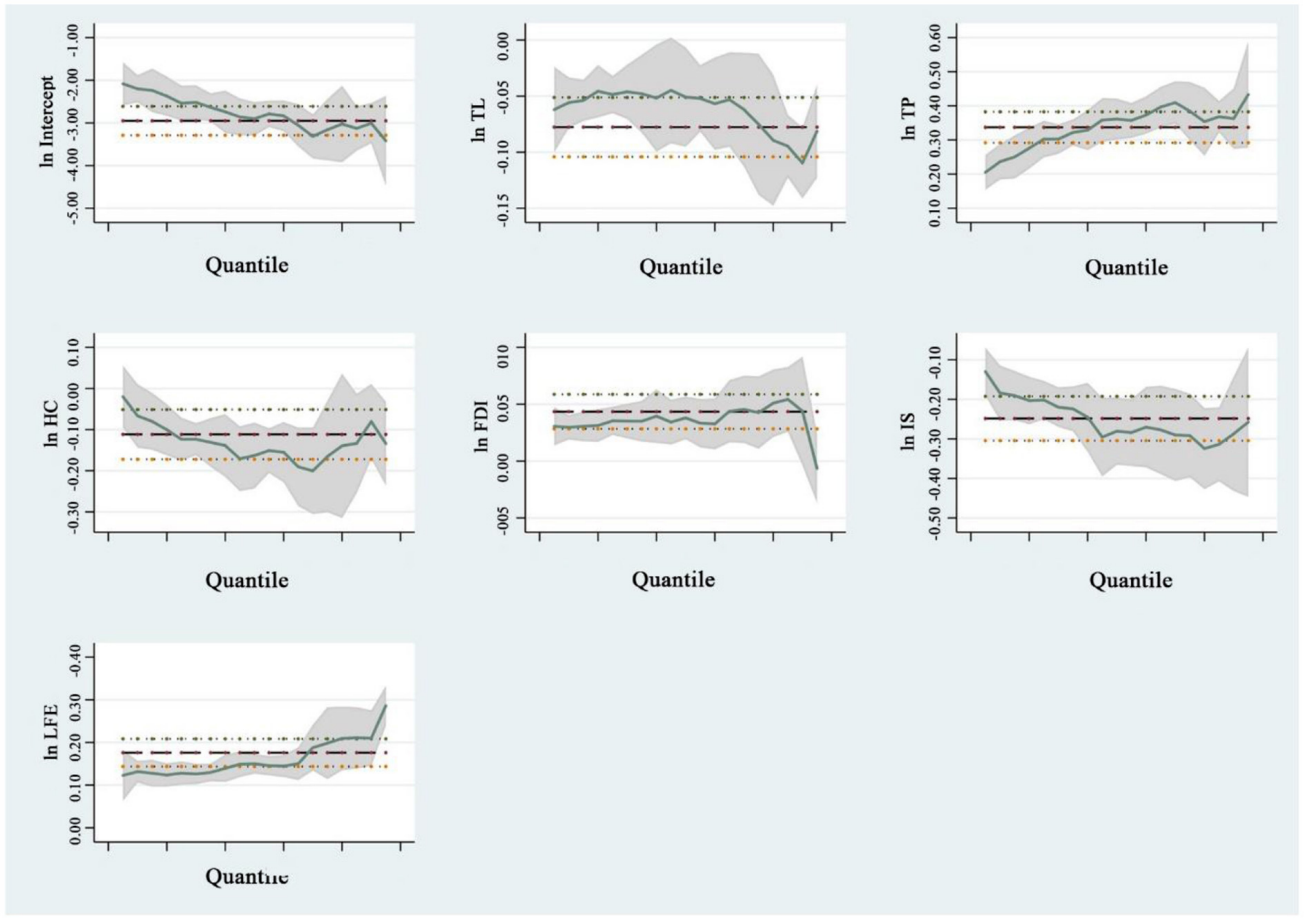
Distribution of the regression elasticity coefficient of influencing factors of the environmental pollution index (EP) in China.

Existing research indicates that the degree of EP differs at various stages of economic development, and the impact of different stages of TL on the EP varies as well. Compared with the ordinary least square regression, the QR model gave a novel perspective on the basis of compensating for the biases and outliers in the data, which could not fulfill the presupposition of mean regression: when the independent variable was determined, more data were mined at different levels of the dependent variable to effectively portray the dynamic relationship between different and dependent variables. Therefore, this study estimated the dynamic relationship between the EP and TL using equations (10) and (11). There was potential for multicollinearity among variables. Based on the QR, the process was as follows: take the logarithm of the primary term of the control variable, conduct a multicollinearity test, eliminate variables with possible collinearity, and finally retain TL, TP, HC, FDI, IS, and LFE. To compare the mean regression coefficient of the traditional panel data model, the OLS model was initially generated. To enhance the evaluation effect, this study utilized nine quantile indexes (i.e., 0.10, 0.20, 0.30, 0.40, 0.50, 0.60, 0.70, 0.80, and 0.90) to evaluate the relationship between various influencing factors and the distribution of the EP under variable conditions. Table 5 lists the panel QR results for different variables, whereas Figure 5 depicts the elasticity coefficient distribution for various variables.

**Table 5:** Least squares method (OLS) and quantile regression (QR) estimation results.

**Variable**	**OLS**	**QR**								
		**0.10**	**0.20**	**0.30**	**0.40**	**0.50**	**0.60**	**0.70**	**0.80**	**0.90**
ln TL	−0.078^***^	−0.056^***^	−0.046^***^	−0.046^***^	−0.052^**^	−0.051^**^	−0.057^***^	−0.062^**^	−0.089^***^	−0.110^***^
	(0.013)	(0.012)	(0.01)	(0.011)	(0.016)	(0.017)	(0.018)	(0.024)	(0.026)	(0.024)
ln TP	0.337^***^	0.236^***^	0.276^***^	0.303^***^	0.329^***^	0.361^***^	0.373^***^	0.410^***^	0.354^***^	0.362^***^
	(0.023)	(0.022)	(0.018)	(0.019)	(0.027)	(0.03)	(0.032)	(0.042)	(0.045)	(0.041)
ln HC	−0.112^***^	−0.067^*^	−0.100^***^	−0.123^***^	−0.139^***^	−0.163^***^	−0.155^***^	−0.200^***^	−0.139^*^	−0.080^*^
	(0.03)	(0.029)	(0.024)	(0.025)	(0.036)	(0.041)	(0.042)	(0.057)	(0.06)	(0.055)
ln FDI	0.044^***^	0.030^***^	0.031^***^	0.035^***^	0.040^***^	0.038^***^	0.033^***^	0.045^***^	0.051^***^	0.044^**^
	(0.007)	(0.007)	(0.006)	(0.006)	(0.009)	(0.01)	(0.01)	(0.014)	(0.015)	(0.013)
ln IS	−0.248^***^	−0.184^***^	−0.203^***^	−0.220^***^	−0.245^***^	−0.281^***^	−0.270^***^	−0.290^***^	−0.324^***^	−0.287^***^
	(0.028)	(0.027)	(0.022)	(0.023)	(0.034)	(0.038)	(0.039)	(0.052)	(0.055)	(0.051)
ln LFE	0.176^***^	0.132^***^	0.124^***^	0.126^***^	0.140^***^	0.150^***^	0.145^***^	0.188^***^	0.209^***^	0.210^***^
	(0.016)	(0.015)	(0.013)	(0.013)	(0.019)	(0.022)	(0.022)	(0.03)	(0.032)	(0.029)
_cons	−2.951^***^	−2.200^***^	−2.372^***^	−2.519^***^	−2.739^***^	−2.903^***^	−2.834^***^	−3.315^***^	−3.025^***^	−3.006^***^
	(0.172)	(0.164)	(0.138)	(0.141)	(0.206)	(0.23)	(0.239)	(0.319)	(0.336)	(0.31)

Values in parentheses are *t-*test values;

***, **, and * represent significance at a confidence level of 1, 5, and 10%, respectively.

Table 5 demonstrates that the regression coefficients (regression curve slopes) of different variables in different quartiles vary; that is, the degree of influence and the effect of different variables on the marginal effects for EP in various quartiles differ. From the perspective of core explanatory variables, regardless of OLS or QR, the impact of TL on the EP was negative at the level of effective significance, and the overall trend initially increased and then decreased with the increase in quantile, indicating that TL had a negative impact on the EP. This indicated that all provinces in the country were in the initial stage of cost saving and that TL support, such as technology improvement and management mode optimization, was poor. Innovation compensation could hardly compensate for the high production costs caused by environmental regulation, and matching funds and policies could hardly demonstrate the impact of environmental pollution levels, making it difficult to offset the negative impact of TL on the crowding-out effect of innovation input. The results showed that TL was the primary contributor to the EP, and that the inhibitory effect of TL on EP was more significant in the middle quartile provinces than that in the high quartile provinces.

Regarding the control variables, the TP, HC, FDI, IS, LFE, all had a significant impact on the EP at the effective significance level. Specifically, the TP had a significant positive correlation with the EP, and the positive effect fluctuated with an increase in the quantile, which generally exhibited a trend of “initially increasing and then decreasing.” The specific realization was that the regression coefficient exhibited an ascending trend in the low and middle quartiles (0.10–0.70) and a descending trend in the high quartiles (0.80–0.90). This showed that increasing the total population would aggravate the level of environmental pollution to a certain extent, because population growth would result in the aggregation and development of various industrial resources, which will aggravate the degree of environmental pollution to a certain extent.

HC had a significant inhibitory effect on the EP, which fluctuated as the quartile increased. Specifically, the regression coefficient revealed an increasing trend in the low and middle quartiles (0.10–0.50), followed by a fluctuating trend in the high quartiles (0.60–0.90). This was because HC reflected the educational level and consciousness of environmental protection of local populations, which is conducive to regional environmental protection.

FDI had a significant positive effect on the EP at the middle and low quartile levels, with a clear inverted “m” - shaped rising trend with an increase in the quartile. This indicated that the influence of FDI on regions with a low EP was evident, whereas the effect of FDI on regions with a high EP was weaker.

IS had a significant inhibitory effect on the EP, which fluctuated with an increase in quantiles. Particularly, the regression coefficient revealed an ascending trend in the low and middle quartiles (0.10–0.50), followed by a fluctuating trend in the high quartiles (0.60–0.90). This indicated that the inhibitory effect of IS in regions with a low EP was minimal, whereas in regions with a high EP, the inhibitory effect of increased IS was amplified.

The positive effect of local LFE on the EP fluctuated with an increase of the quantile. Generally, it showed an “*n*” type fluctuating trend with an increase of the quantile. The specific performance is that in the low and middle quantiles (0.10–0.60), the regression coefficient was low, and the positive effect on the EP was weak. Moreover, it was at a high level in the high quartile (0.70–0.90), and it had a strong positive effect on the EP. This demonstrated that the regression coefficient of LFE increased as the quantile increased. The positive effect of LFE gradually increased, indicating that the degree of environmental pollution could effectively be reduced by increasing LFE during the transition process of provincial EP from low to high.

The aforementioned results demonstrated that each variable had different effects on the EP at various quantile levels. On this basis, the confidence interval diagram of the QR curve of the various quantiles was produced to investigate the impact of different variables on the EP (Figure 5). The regression elasticity coefficient distribution of variables demonstrated that the influence of social and economic factors on the level of green TL was phased. The upper and lower limits of OLS estimation coefficients and their confidence intervals are represented as horizontal lines, and their coefficients and confidence intervals remain unchanged as quantiles change. For the regression coefficient of the QR model, with a change of quantile conditions (Figure 5), it was discovered that the regression coefficient significantly changed with a high EP.

The TP, HC, FDI, IS, and LFE had a significant influence on the EP at the effective significance level, regardless of whether OLS or QR was used. In particular, the regression coefficients and confidence intervals of the TP, FDI, and LFE were all >0, indicating a positive effect on the EP. The confidence intervals of the TP and FDI were gradually widening, indicating that the standard deviation of the coefficient and its volatility were gradually increasing.

The promoting effect of TP and TS on the EP of low and middle quartile provinces was greater than that of high quartile provinces; FDI had a more substantial role in promoting the improvement of the EP of high-ranking provinces than other variables. The regression coefficients and confidence intervals for TL, HC, and IS were < 0, indicating a negative impact on the EP.

Furthermore, the coefficient estimates for different quantiles of each variable fell outside the coefficient confidence interval of the mean regression model, indicating that the mean regression model was partially irrational and the QR model could better explain the relationship between variables. However, compared to the results of panel QR, the TP, HC, FDI, IS, and LFE estimated by OLS fixed-effect regression were the same at the level of effective significance, regardless of whether it was OLS or QR.

## Conclusion and policy recommendations

This study analyzes the spatial distribution pattern of the national EP and TL, explores its spatiotemporal evolution trend, and then quantitatively evaluates the effect of the influencing factors on the EP. It ensures a thorough understanding of the specific mechanisms of inter-provincial TL capacity and environmental pollution in China, as well as promoting the formulation of innovative incentive policies and environmental pollution control measures that are reasonable and effective by the Chinese government. This study found there is a significant spatial autocorrelation between the EP and TL. Overall, the spatial distribution of the EP was directional, being concentrated in the southwest and northeast. TL showed a “north-south change, high in the east, and low in the west” trend. Regional differences and the phenomenon of polarization were clearly visible. Regardless of using OLS or QR, TL, HC, IS all had a constraining effect on the EP at the effective significance level, whereas the TP, FDI, and LFE were positively correlated to the EP.

Certainly, there were some shortcomings in this study. Factors were used which were difficult to quantify, such as environmental awareness and policy control, which may affect the research results. Future research should use appropriate methods to incorporate the aforementioned factors into the analysis of influencing factors, enhancing the accuracy. In additional, the research scale should be more refined.

Based on the findings above, the following recommendations can be proposed: Firstly, we should completely understand the complex relationship between TL and EP, and then we should formulate a positive strategic plan for green industrial development. We should maximize the role of TL in industrial production and increase investment in green technology R&D to advance environmental protection technologies. Secondly, in the context of high-quality economic development, we should actively encourage the optimization and upgrading of the industrial sector, especially the secondary and tertiary industries. To achieve the optimization and upgrading of the IS during industrialization. Thirdly, we should strengthen policy support and environmental regulation for the development of regional TL by enhancing the green policy framework. Finally, we should optimize the market competition environment, strengthen the legislative framework for the innovation of green technology by enterprises, and enhance the system of environmental regulations and standards.

## Data availability statement

The raw data supporting the conclusions of this article will be made available by the authors, without undue reservation.

## Author contributions

CW and XG: conceptualization, methodology, writing—review, editing, and formal analysis. CW: software, investigation, writing—original draft preparation, and data curation. All authors have read and agreed to the published version of the manuscript.

## Funding

The GDAS' Project of Science and Technology Development (Grant number: 2022GDASZH-2022010202), and the Science and Technology Program of Guangdong (Grant number: 2021B1212100006).

## Conflict of interest

The authors declare that the research was conducted in the absence of any commercial or financial relationships that could be construed as a potential conflict of interest.

## Publisher's note

All claims expressed in this article are solely those of the authors and do not necessarily represent those of their affiliated organizations, or those of the publisher, the editors and the reviewers. Any product that may be evaluated in this article, or claim that may be made by its manufacturer, is not guaranteed or endorsed by the publisher.
